# Gene expression and enzyme activity analysis of carbohydrate digestion in *Strongylocentrotus purpuratus* larvae

**DOI:** 10.1242/jeb.250125

**Published:** 2025-05-08

**Authors:** Jasper Hildebrand, Meike Stumpp

**Affiliations:** Zoological Institute, Christian-Albrechts University of Kiel, 24118 Kiel, Germany

**Keywords:** Glycosidases, Digestive enzymes, Laminarin, Amylase, Xylan, Trehalose, Cellulose, Sea urchin

## Abstract

Carbohydrates play multifaceted roles in marine ecosystems, serving as structural components in algae, energy storage molecules and vital nutrients for marine organisms. The purple sea urchin, *Strongylocentrotus purpuratus*, undergoes metamorphosis during ontogeny, transitioning its feeding strategy from microalgae to macroalgae as the primary food source. However, the digestive mechanisms underlying carbohydrate digestion in sea urchin larvae remain poorly understood. We investigated the carbohydrate digestion capabilities of *S. purpuratus* larvae, using expression-level analysis of candidate genes putatively involved in carbohydrate digestion, quantification of enzyme activity, and pH and temperature optima characterization for the digestion of starch, laminarin, cellulose, xylan and trehalose. Transcriptomic analyses revealed the expression of genes encoding putative carbohydrate-degrading enzymes during early larval development. RT-qPCR demonstrated age- and/or feeding-dependent expression patterns of glycosidase candidate genes *β-1,3-glucanase* (*laminarinase*), *α-amylase*, *endo-β-1,4-glucanase D-like* (*cellulase*), *xylanase*/*β-glucanase-like* and *trehalase*. Furthermore, enzymatic assays elucidated differential temporal patterns, and thermal and pH optima of associated carbohydrate-degrading enzymes. A comparison of the enzymatic degradation of five substrates demonstrated that laminarinase activity was five times higher than the activity of enzymes involved in digesting starch, cellulose, xylan and trehalose, leading to a hypothesis regarding the importance of laminarin for larval growth.

## Teaser

**Summary:** Investigation of the carbohydrate digestion capabilities of *Strongylocentrotus purpuratus* larvae reveals comparably high digestive capacity for laminarin, suggesting it may be an important carbon source in benthic and planktonic communities

## INTRODUCTION

Polysaccharides, high molecular weight carbohydrates, are the major form of photosynthetically assimilated carbon in the biosphere and can be broadly categorized into one of two classes: homopolysaccharides and heteropolysaccharides. Homopolysaccharides (e.g. starch, glycogen or cellulose) are characterized by a single repeating monosaccharide type. Heteropolysaccharides (e.g. xylan) consist of two or more different monosaccharide units. In the marine environment, carbohydrates have diverse functions, serving not only as structural molecules in plants and algae but also as energy storage molecules important for plants, algae and animals, and consequently for the nutrition of consumers. In marine phytoplankton, the major food source for planktotrophic larvae, carbohydrates act as essential structural components contributing to the integrity of their cells as well as energy storage molecules within the cells (Geider and La Roche, 2002).

Marine microalgae are the basis of marine food webs. With approximately 50 gigatons of annually fixed CO_2_, they contribute to roughly half of the world's primary production (Yang et al., 2020). The proportion of carbohydrates in the dry mass of macroalgae and microalgae varies considerably across different species, ranging for example from 8–14% dry mass in the cyanobacterium *Spirulina platensis* up to 33–64% dry mass in the green algae *Spirogyra* sp*.* (Becker, 2007). Also, their carbohydrate composition itself, e.g. the proportions of laminarin, starch, cellulose and xylan, differs strongly (Markou et al., 2012). Recently, laminarin has been identified as a major carbohydrate component in particulate organic matter in phytoplankton communities (Becker et al., 2020). The variability in microalgal composition leads to a high diversity of carbohydrate resources as potential food sources for herbivorous zooplankton, which is commonly reflected in the patterns of digestive enzymes in marine organisms (Hylleberg Kristensen, 1972).

Sea urchins play a vital role in many marine ecosystems. As herbivores, they regulate algal populations in between states of kelp forests and sea urchin barrens (Pearse, 2006). For several decades, the purple sea urchin, *Strongylocentrotus purpuratus*, has been a model organism within the research areas of marine biology, such as evolutionary, developmental and molecular biology (Ernst, 1997). Sea urchins have a biphasic life cycle with a planktonic larva and a benthic adult stage. When *S. purpuratus* undergoes metamorphosis, the individual transitions from a pelagic to a benthic lifestyle that is consequently accompanied by a shift in their primary food source from planktonic microalgae to macroalgae. With macroalgae and microalgae as the sea urchin's dominant food sources, carbohydrates constitute a significant proportion of the adult and larval diet. Carbohydrates encompass a large group of polysaccharides that differ in their types of linkage (alpha and beta) between sugars as well as in monosaccharide composition (e.g. glucose, xylose, galactose). Depending on the linkage between the monomers in polysaccharides, different enzymes are needed to break down carbohydrates into their components. In marine diets, there are major nutritionally important carbohydrates derived from algae or plants, such as laminarin, starch, cellulose, xylan and trehalose.

Laminarin, a predominantly β-1,3-glucan homopolysaccharide, serves dual functions. It is an important carbon source for zooplankton species, contributing up to 50% to the particulate organic carbon in coastal waters (Becker et al., 2020), and it is the major structural component in *Laminaria* sp. (Black, 1950), one of the main food sources for adult *S. purpuratus*. Laminarin-degrading enzymes, such as β-1,3-glucanase (laminarinase), are present in many marine invertebrates, such as crustaceans, mollusks, bivalves and tunicates, as well as in adult sea urchins (Sova et al., 1970) and their larvae (Vacquier, 1971). Starch is a glucan homopolysaccharide, which is present in virtually all marine phototrophic organisms. It is hydrolyzed by amylases, which are ubiquitously found in the digestive tracts of marine animals. Cellulose, a β-1,4-glucan homopolysaccharide, is recognized as an important biopolymer in nature and is present in cell walls of plants in a variety of photosynthetic organisms (Kamide, 2005). Putative genes for cellulases were found in *Strongylocentrotus nudus* (Nishida et al., 2007) and *Strongylocentrotus intermedius* (Hasegawa et al., 2012). Xylan, a heteropolysaccharide, serves as another important constituent in plant cell walls, representing the most abundant hemicellulose, and has also been well described in macroalgae (Hsieh and Harris, 2019) and in some (Domozych, 2019) but not all microalgae (Mussgnug et al., 2010). Trehalose, a non-reducing α-1,1-disaccharide, is widespread in invertebrates, bacteria, fungi and plants, but is absent in vertebrates (Shukla et al., 2015). Trehalase is the only enzyme known to catalyze the breakdown of trehalose into two d-glucose molecules under physiological conditions (Terra and Ferreira, 2005).

Adult sea urchins possess the ability to digest structural carbohydrates such as laminarin, cellulose and xylan; however, there has been a decades-long debate about whether the degradation of cellulose (Nishida et al., 2007) or laminarin (Vacquier, 1975) is microbially assisted. To date, comparative studies on carbohydrate digestion in sea urchin larvae are scarce. We therefore aimed at elucidating aspects of carbohydrate digestion in *S. purpuratus* larvae. We identified candidate genes with a putative function in carbohydrate digestion for the aforementioned carbohydrates and quantified their expression in response to high and low levels of food. Furthermore, we quantified carbohydrate-digesting enzyme activities (laminarinase, amylase, cellulase, xylanase and trehalase) in crude extracts of larvae raised with high and low food using specific substrates and established pH and thermal profiles.

## MATERIALS AND METHODS

### Adult and larval cultures

Adult *Strongylocentrotus purpuratus* (Stimpson 1857) specimens were obtained from the Californian coast (La Jolla, CA, USA) and subsequently transported to the Helmholtz Center for Ocean Research Kiel (GEOMAR). The organisms were housed in recirculating systems filled with seawater from the Kiel Fjord, supplemented with artificial sea salt (Pro Reef, Tropic Marin, Hünenberg, Switzerland) to achieve a salinity of 31.5 psu. One-third of the water in the system was regularly replaced 2–3 times per week and the sea urchins were provided with a continuous supply of kelp (*Macrocystis pyrifera*) 3 times weekly.

To induce spawning for larval cultivation, adult sea urchins were gently shaken. Eggs were collected in 0.2 μm filtered seawater (FSW) from the Kiel Fjord, which was supplemented with artificial sea salt to a salinity of 31.5 psu, and briefly rinsed before fertilization. Dry sperm was directly obtained and used to fertilize the eggs by mixing. Fertilization success was always above 98% and embryos were monitored until the 2-cell stage before they were transferred into 2 l culture flasks at an initial concentration of 12 larvae ml^−1^.

Cultures were maintained under low light conditions, following a 12 h dark:12 h light cycle at 15°C, with continuous mixing facilitated by a gentle stream of pressurized air bubbles. Larval health was assessed by sampling two 10 ml water samples every 2 days. Larvae were visually inspected for morphology and counted to assess mortality (Fig. S4). Water exchanges were performed every second day, and larval feeding occurred after the water change, commencing at 3 days post-fertilization (dpf). Larvae were subjected to two distinct feeding conditions: under low food conditions, they were provided with 500 cells ml^−1^ of *Rhodomonas* sp. every other day (immediately after the water change); for high food conditions, they were provided with 8000 cells ml^−1^. The low food treatment was sufficient to ensure survival and basic health during the 10 day experiment, whereas the high food treatment resulted in residual algal cells always being visible before water changes. Pilot experiments demonstrated different feeding levels with maximum larval feeding rates above 8000 cells ml^−1^ and significant differences compared with larvae reared under 500 cells ml^−1^ (Supplementary Materials and Methods and Fig. S5). For each food treatment, 3 or 4 larval cultures were prepared and treated as biological replicates. At 3, 5, 7, 9 and 10 dpf, larval body length was measured for both feeding conditions. Seawater characteristics, such as salinity, temperature and pH, were monitored every second day after water changes (Table 1) using a handheld meter (WTW pH-Meter 3110 Set 2 and Conductometer WTW Cond 3110 probes, Xylem Analytics, Weilheim, Germany).

**Table 1.** Experimental water chemistry in *Strongylocentrotus purpuratus* larval experiments

*Rhodomonas* sp. (cells ml^−1^)	Ontogeny (Fig. 1)		Temperature/pH characterization (Figs 4 and 5)
	500	8000	8000
Salinity (psu)	31.5±0.1	31.4±0.1	31.4±0.3
Temperature (°C)	15.3±0.2	15.3±0.2	15.5±0.5
pH_NBS_	8.25±0.03	8.28±0.02	8.22±0.05
Sampling points (dpf)	3, 5, 7, 9, 10		7
Experimental length (days)	10		7

Salinity, temperature and pH_NBS_ were assessed every other day throughout the experimental duration. The presented values represent means±s.d. For RT-qPCR samples, *n*=4; for temperature/pH characterization and ontogeny samples, *n*=3. dpf, days post-fertilization.

### Sampling for enzyme and RT-qPCR assays

Samples were collected as previously described (Hildebrand et al., 2023). Each sample consisted of approximately 7500 larvae. After removing excess water, the samples were promptly frozen in liquid nitrogen and subsequently stored at −80°C until further processing. For this study, two experiments were performed: one for candidate gene expression and one for enzymatic assays.

For RT-qPCR analysis and for comparison with our previous study on protein digestion (Hildebrand et al., 2023), putative glycosidase expression data were generated from the same cDNA samples used in our companion study (*n*=4, experiment 1 of Hildebrand et al., 2023). However, as enzyme measurements require large numbers of larvae (minimum 5000 larvae per sample), an additional experiment was conducted to obtain sufficient enzyme samples for carbohydrate digesting enzyme analysis. Thus, to characterize enzymatic activities at different larval ages and under varying pH and temperature conditions, larvae from the specified ages or from 7 dpf (for pH and temperature assays) were used (*n=*3). Note that samples for enzymatic analysis and RT-qPCR were taken from cultures originating from two separate fertilizations, maintained under the same maintenance protocol.

### RT-qPCR and single cell transcriptomic analyses

RT-qPCR analysis was performed following established procedures as outlined in prior work (Hu et al., 2018). In summary, cDNA samples from our companion paper (Hildebrand et al., 2023) were used. RT-qPCR analysis was performed using the 2X qPCRBIO SyGreen Mix Hi-ROX (PCR Biosystems Ltd, London, UK) in a two-step RT-qPCR process. Five primer pairs (Table 2) were designed using NCBI Primer-BLAST, targeting exon junctions of candidate genes with a putative function in carbohydrate digestion, using mRNA sequences obtained from the *S. purpuratus* genome database echinobase.org (Arshinoff et al., 2022). Candidate genes were chosen based on their transcription patterns during early development, with increasing expression towards digestive tract formation. Primer specificity was validated through standard PCR, confirming the amplification of a single, specific product within the expected size range. RT-qPCR was performed on a QuantStudio 1 Real-Time PCR Instrument (Applied Biosystems, Waltham, MA, USA). The cycling conditions included an initial denaturation step for 10 min at 95°C, followed by 40 cycles of 15 s at 95°C, 20 s at 58°C, and 35 s at 72°C, ending with a melting curve analysis. Quantification utilized the ΔΔCT method, and gene expression levels were normalized to those of the stable housekeeping gene *EF1a* (SPU_000595), as previously validated throughout ontogeny (Hu et al., 2020).

**Table 2.** Primer data for RT-qPCR (ID 1–5) for five digestive glycosidase candidate genes of *S. purpuratus*

ID	Primer name	Corresponding gene	Primer sequence (5'–3′)	Amplicon length	NCBI accession no.
1	glu qF1	*β-1,3-glucanase* (*laminarinase*) (LOC373274)	ACTCGACATGCTGAACGGAG	132 bp	XM_011671772.2
	glu qR1		GGGCCAAGAAACCTTTCACA		
2	amy qF1	*α-amylase 1* (LOC581100)	GGTTGAAAGCAGAGGTAACGC	160 bp	XM_030980519.1
	amy qR1		TCCGAAAGTTTGTCCCTCGG		
3	end qF1	*endo-β-1,4-glucanase D-like* (*cellulase*) (LOC115928076)	TCAAAACGCTTGAAATGCATGA	174 bp	XM_030994773.1
	end qR1		CCAAAATACATTGCTACTCTGGGA		
4	xyl qF1	*xylanase/β-glucanase-like* (LOC105443845)	GGAGATTCGATCGACCTACCG	152 bp	XM_030986626.1
	xyl qR1		GGCTCAAGTACCGGTTCGAT		
5	tre qF1	*trehalase* (LOC580425)	CGCCATGCGCTAGTTCAATC	164 bp	XM_030977761.1
	tre qR1		ATTCGCATCCGTCCGATCAA		
6	EF1a qF	*translation elongation factor EF1a* (LOC548620)	CCGACCTTGGAAAGGGATCG	194 bp	NM_001123497.2
	EF1a qR		ACAGTCGGCCTGTGTGAGGTTC		

*EF1a* was used as a reference gene for RT-qPCR normalization. Primer efficiency was always >95%.

Candidate genes with a putative function in carbohydrate digestion were screened for their cellular expression patterns using single-cell transcriptome data from early pluteus larvae. Single-cell data were kindly provided by Jonas Brandenburg and David Garfield (Integrative Research Institute for the Life Sciences, Humboldt-Universität zu Berlin, Germany) who conducted the experiments and analysis as published (Chang et al., 2021). In brief, pluteus stage larvae were incubated for 5 min in hyalin extraction medium (0.3 mol l^−1^ glycine, 0.3 mol l^−1^ NaCl, 0.01 mol l^−1^ KCl, 0.01 mol l^−1^ MgSO_4_, 0.01 mol l^−1^ Tris, pH 8.0, and 0.002 mol l^−1^ EGTA, pH 8.2) on ice. Cell dissociation was achieved by gently pipetting until >95% single cell suspension was obtained. The dissociated single cells were washed twice in calcium-free seawater (CFSW; 454 mmol l^−1^ NaCl, 9 mmol l^−1^ KCl, 48 mmol l^−1^ MgSO_4_, 6 mmol l^−1^ NaHCO_3_, pH 8.2) and assessed for viability (>95%) using propidium iodide staining, followed by fixation in 90% ice-cold methanol. The fixed cells were rehydrated in CFSW containing 0.1% BSA (w/v) and an RNase inhibitor (SUPERaseIN, Thermo Fisher Scientific). Subsequent processing followed the 10xGenomics Single Cell 3′ version 3 user guide. Library sequencing was performed using an Illumina Hiseq system (Illumina, San Diego, CA, USA), yielding an average of 23,015 raw reads per cell barcode. Raw reads underwent demultiplexing, alignment to the *S. purpuratus* genome (Sodergren et al., 2006), and counting using the CellRanger pipeline from 10xGenomics (version 3.0.2). Barcodes that did not meet the criteria, with fewer than 1000 reads, fewer than 500 expressed genes or more than 25% mitochondrial reads, were excluded to retain high-quality cells. Basic uniform manifold approximation and projection (UMAP) dimensional reduction and clustering were executed using the Seurat version 3.2 workflow (Butler et al., 2018).

### Enzyme sample preparation

The crude enzyme extract was prepared following previously established procedures (Hildebrand et al., 2023). Briefly, frozen larvae (approximately 7500 per sample) were re-suspended in 550 μl of nuclease-free water and homogenized on ice using an ultrasonic cell disruptor (Bandelin Sonoplus HD 2200, Berlin, Germany) for six cycles of 15 s, with a 15 s pause between each cycle. The resulting homogenates were centrifuged for 15 min at 21,000 ***g*** and 4°C. The supernatants were carefully transferred into new reaction tubes and kept on ice until further use. The protein concentration was quantified using a Micro BCA Protein Assay Kit (Thermo Scientific, Waltham, MA, USA) with bovine serum albumin as the standard, following the manufacturer's instructions.

### Enzymatic characterization

The enzymatic activity for degrading carbohydrates across substrates, including starch, laminarin, cellulose, xylan and trehalose, was quantified using 3,5-dinitrosalicylic acid (DNS) as a dye to detect reducing sugar ends (Miller, 1959). Degradation activities were quantified by measuring the rate of reducing sugar production from the hydrolysis of laminarin (Prod.#L9634), soluble starch (Prod.#S9765), carboxymethylcellulose sodium salt (Prod.#C4888), xylan (Prod.#8659.1) and d-(+)-trehalose dihydrate (Prod.#T9531; all sourced from Merck, Darmstadt, Germany, except xylan, which was obtained from Roth, Karlsruhe, Germany). For the enzymatic reactions, 200 μl of 1% (w/v) substrate solution in 0.1 mol l^−1^ acetate buffer (pH 5.0, or pH 6.0 for starch degradation) was mixed with 10 μl of the crude enzyme extract. Negative controls were prepared by replacing the crude enzyme extract with 50 μl of water. To determine initial (*t*_0_) values, 50 μl of each reaction was directly diluted with 50 μl of 1% (w/v) DNS solution (10 g l^−1^ 3,5-dinitrosalicylic acid, 30 g l^−1^ sodium potassium tartrate, 16 g l^−1^ sodium hydroxide) and boiled at 100°C. Incubation was carried out with the remaining reaction mixtures for 18 h at 30°C with gentle shaking (300 rpm). For the final stop reaction (*t*_1_), again 50 μl of each reaction was diluted with 50 μl of 1% (w/v) DNS solution and boiled at 100°C. The amount of reducing sugars produced in each reaction was determined spectrophotometrically at 540 nm, using the difference between the positive reactions and negative controls for *t*_0_ and *t*_1_ and, finally, the difference *t*_1_−*t*_0_ was standardized using glucose (xylose for xylan characterization) and set in relation to the amount of protein used and the reaction time, so that the results are presented in pmol reducing sugars mg^−1^ protein h^−1^.

pH optima for degradation of different carbohydrates were determined using 0.1 mol l^−1^ citrate buffer (pH 3.0, 4.0, 5.0, 6.0), 0.1 mol l^−1^ Tris-HCl buffer (pH 7.0, 8.0, 9.0) and 0.08 mol l^−1^ glycine-NaOH buffer (pH 10.0). Optimal temperatures for enzymatic activity were determined by incubating reactions across a range from 10 to 80°C, using 0.1 mol l^−1^ citrate buffer, pH 5.0 (or pH 6.0 for starch degradation). Enzyme thermostability was assessed by measuring residual activity after preincubating the crude enzyme extract for 1 h within the temperature range of 10–80°C. The subsequent reaction occurred under standard conditions [0.1 mol l^−1^ citrate buffer, pH 5.0 (or 6.0 for starch) and at 30°C].

### Statistical analyses

Figures and statistical analyses were performed using GraphPad Prism 8.4.3. Biological replication was defined as the number of larval cultures (*n=*3 cultures for enzymatics and *n=*4 cultures for qPCR) within a food treatment (high and low food with 3 or 4 cultures each, respectively). Prior to statistical evaluation, datasets were assessed for normality (Kolmogorov–Smirnov test). The obtained results were then analyzed using one-way ANOVA followed by Tukey's multiple comparisons test, repeated measures ANOVA and non-linear regression. Statistical significance was defined as *P*<0.05. Statistics are included in the text and in Table S1.

## RESULTS

### Larval cultures

For the abiotic parameters of the cultures for the RT-qPCR samples (*n*=4), see Hildebrand et al. (2023). The abiotic parameters all cultures of this study remained consistently within a narrow range (Table 1). Salinity fluctuated between 31.4±0.3 psu and 31.5±0.1 psu. Temperature ranged from 15.3±0.2 to 15.5±0.5°C, while pH_NBS_ varied from 8.22±0.05 to 8.28±0.02 (*n*=3). Food concentration did not significantly influence larval body length over the full duration of cultivation (*P*=0.6839, *t*=0.4192, d.f.=10; Fig. S3A). Culture density loss was less than 20% of initial density (Fig. S3B).

### Screening of potential glycosidases in *S. purpuratus* larvae

Candidate genes encoding putative carbohydrate-degrading enzymes [*β-1,3-glucanase* (*laminarinase*) (LOC373274); *α-amylase 1* (LOC581100); *endo-β-1,4-glucanase D-like* (*cellulase*) (LOC115928076); *xylanase*/*β-glucanase-like* (LOC105443845); and *trehalase* (LOC580425)] were identified through analysis of the published transcriptomic data (Tu et al., 2014). These candidate genes were expressed within the initial 72 h post-fertilization, coinciding with the onset of the functional larval digestive system, suggesting their involvement in digestion (Fig. S1). In-depth single-cell transcriptomic analyses from pluteus-stage larvae 72 h post-fertilization revealed distinctive expression patterns of putative carbohydrate-degrading enzymes in different cell types (Fig. S2). However, none of the gene sequences used here has been validated with functional genomic approaches to confirm their function in larval digestion. *β-1.3-glucanase* (*laminarinase*) was expressed in pancreatic-like cells and scattered within midgut cells (Fig. S2A). *α-amylase 1* had negligible expression levels in midgut and pancreatic-like cells (Fig. S2B). There was also negligible signal for *endo-β-1,4-glucanase D-like* (*cellulase*) outside ecto-aboral cell types, where the signal was notably low (Fig. S2C). The *xylanase/β-glucanase-like* signal was in filopodial cells (Fig. S2D), showing a specialized expression pattern within the cellular landscape, while the *trehalase* signal manifested in both midgut and hindgut-associated cells (Fig. S2E), underlining its versatile presence.

**Fig. 1.**
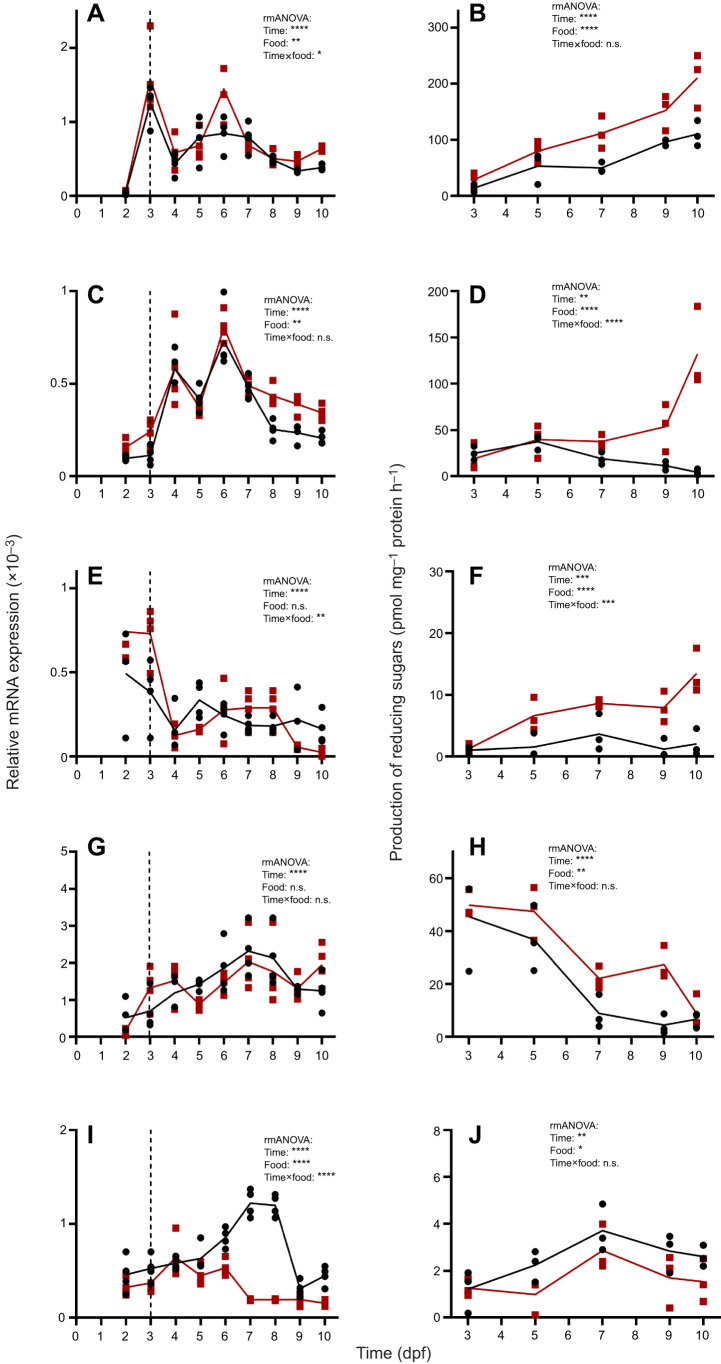
**Ontogeny of mRNA expression (left) and enzymatic activity (right) of carbohydrate-degrading enzymes in *Strongylocentrotus purpuratus* under different feeding conditions.** Enzymatic assays were conducted at 30°C and pH 5 (or pH 6 for starch degradation); reactions were stopped and the amount of released reducing sugar equivalents detected with DNS. (A) Relative expression of *β-1,3-glucanase* and (B) laminarin degradation. (C) Relative expression of *α-amylase 1* and (D) starch degradation. (E) Relative expression of *endo-β-1,4-glucanase D-like* and (F) carboxymethylcellulose degradation. (G) Relative expression of *xylanase/β-glucanase-like* and (H) xylan degradation. (I) Relative expression of *trehalase* and (J) trehalose degradation. Two food treatments [500 cells ml^−1^ (black) and 8000 cells ml^−1^ (red) *Rhodomonas* sp.] were administered. Gene expression levels were normalized to those of the housekeeping gene *EF1a* (*n*=4 for gene expression and *n*=3 for enzymatic activity). Repeated measures ANOVA was used to analyze differences depending on larval age, feeding condition and interactions between age and feeding. For statistics, see Results and Table S1. Asterisks indicate significance levels: *P≤0.05, **P≤0.01, ***P≤0.001, ****P≤0.0001; n.s., not significant. Dashed lines indicate the start of larval feeding.

RT-qPCR was employed to assess the impact of larval age (ontogeny) and feeding condition (500 and 8000 cells ml^−1^ *Rhodomonas* sp.) on the mRNA expression of the aforementioned candidate genes. The transcript abundance of the candidate genes differed overall in response to feeding treatment. The mRNA expression of *β-1,3-glucanase* (*laminarinase*) differed significantly (rmANOVA, *F*_8,54_=31.92, *P*<0.0001) with larval age and significantly between the feeding treatments (rmANOVA, *F*_1,54_=8.68, *P*=0.0047). Furthermore, interactions between feeding treatments and larval age (rmANOVA, *F*_8,54_=2.48, *P*=0.023) were observed (Fig. 1A). For *α-amylase 1*, * *gene expression was significantly different over the larval age (rmANOVA, *F*_8,54_=46.49, *P*<0.0001) and between feeding treatments (rmANOVA, *F*_1,54_=15.56, *P*=0.0002), but no interactions were found (rmANOVA, *F*_8,54_=1.76, *P*=0.11; Fig. 1C).

The expression of *endo-β-1,4-glucanase D-like* (*cellulase*) was food independent (rmANOVA, *F*_1,54_=1.33, *P*=0.25), but differed with larval age (rmANOVA, *F*_8,54_=14.58, *P*<0.0001) and there were interactions between these factors (rmANOVA, *F*_8,54_=3.68, *P*=0.0017; Fig. 1E). The same results were found for the expression of *xylanase*/*β-glucanase-like*, where there was no difference between feeding conditions (rmANOVA, *F*_1,54_=0.053, *P*=0.82) and some differences with larval age (rmANOVA, *F*_8,54_=8.59, *P*<0.0001), but there were no interactions between those factors (rmANOVA, *F*_8,54_=1.63, *P*=0.14; Fig. 1G). *trehalase* had the greatest differences in response to food (rmANOVA, *F*_1,54_=172.76, *P*<0.0001) with a striking effect of 63-fold higher expression levels under low food conditions compared with the high food treatment at 7 dpf. Overall *trehalase* transcript abundance differed with larval age (rmANOVA, *F*_8,54_=18.88, *P*<0.0001) and there was a significant interaction between larval age and feeding conditions (rmANOVA, *F*_8,54_=14.58, *P*<0.0001; Fig. 1I).

**Fig. 2.**
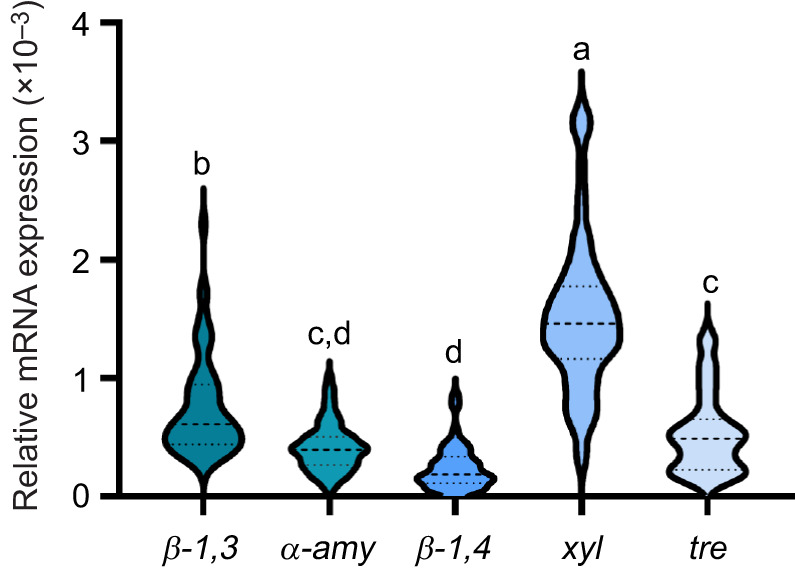
**Comparative representation of candidate gene expression data from analyzed samples.** The data included in this figure (from Fig. 1A,C,E,G,I) pertain to feeding-competent larvae from 3 days post-fertilization onwards, without differentiation based on feeding conditions (*n*=64). Statistical analysis was conducted using one-way ANOVA followed by Tukey's multiple comparisons test. *β-1,3*, *β-1,3-glucanase*; *α-amy*, *α-amylase 1*; *β-1,4*, *endo-β-1,4-glucanase*; *xyl*, *xylanase/β-glucanase-like*; *tre*, *trehalase.* Different letters indicate statistically significant differences (one-way ANOVA, *F*_4,315_=15.03, *P*<0.0001).

Independent of feeding treatment or larval age, *xylanase/β-glucanase-like* with 1.5(±0.64)×10^−3^, followed by *β-1,3-glucanase* (*laminarinase*) with 7.4(±4.1)×10^−4^, had the highest mean transcript levels relative to *EF1a*. *α-amylase 1* and *endo-β-1,4-glucanase D-like* (*cellulase*) had the lowest mean transcript levels with 4.1(±2.0)×10^−4^ and 2.3(±1.8)×10^−4^, respectively (Fig. 2).

### Differential temporal patterns in glycosidic activity during larval ontogeny

The enzymatic production of reducing sugars from the substrates laminarin, starch, carboxymethylcellulose, xylan and trehalose was measured at different time points using the DNS method. The degradation of four of the five substrates examined (i.e. laminarin, starch, cellulose and trehalose) increased with larval age. There were differences between the two food conditions, with higher activity in the high food treatment samples for three of the five substrates (i.e. laminarin, starch and cellulose) especially in older larvae. Specifically, activity was 91% higher for laminarin, 2961% higher for starch and 559% higher for cellulose in high feeding conditions compared with low feeding conditions at 10 dpf (Fig. 1).

The production of reducing sugars with laminarin as a substrate differed significantly with larval age (rmANOVA, *F*_4,20_=27.35, *P*<0.0001) and between feeding conditions (rmANOVA, *F*_1,20_=32.22, *P*<0.0001) with no interactions between these factors (rmANOVA, *F*_4,20_=2.66, *P*=0.06; Fig. 1B). There was a comparable production of reducing sugars using starch as a substrate and production was not only significantly different under different food conditions (rmANOVA, *F*_1,20_=29.88, *P*<0.0001) but also with larval age (rmANOVA, *F*_4,20_=5.69, *P*=0.0032) and with interactions between feeding and age (rmANOVA, *F*_4,20_=12.70, *P*<0.0001; Fig. 1D). Using carboxymethylcellulose, enzymatic activity differed significantly depending on larval age (rmANOVA, *F*_4,20_=7.69, *P*=0.0006) and different feeding conditions (rmANOVA, *F*_1,20_=51.42, *P*<0.0001), with interactions between these factors (rmANOVA, *F*_4,20_=5.09, *P*=0.0054; Fig. 1F). For xylan and trehalose as a substrate, the same results were found with differences in enzyme activity depending on larval age (rmANOVA, *F*_4,20_=25.03, *P*<0.0001 and *F*_4,20_=5.23, *P*=0.0048) and feeding conditions (rmANOVA, *F*_1,20_=11.20, *P*=0.0032 and *F*_1,20_=7.91, *P*=0.0107) but no interactions between these factors were found (rmANOVA, *F*_4,20_=1.32, *P*=0.30; Fig. 1H; and *F*_4,20_=0.60, *P*=0.67; Fig. 1J).

**Fig. 3.**
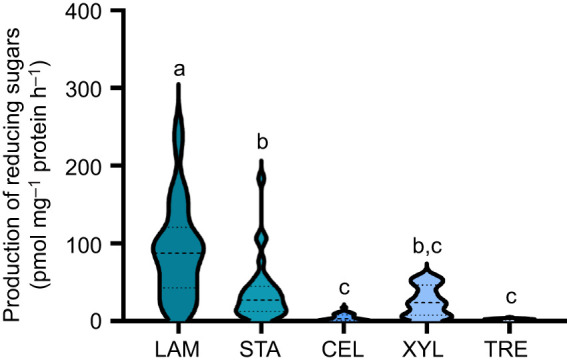
**Comparative representation of produced reducing sugar equivalents from analyzed samples.** The data included in this figure (from Fig. 1B,D,F,H,J) pertain to feeding-competent larvae from 3 days post-fertilization onwards, without differentiation based on feeding conditions (*n*=30). LAM, laminarin; STA, starch; CEL, carboxymethylcellulose; XYL, xylan; TRE, trehalose. Different letters indicate statistically significant differences (one-way ANOVA, *F*_4145_=34.65, *P*<0.0001).

When comparing the activity of all glycosidase groups investigated irrespective of food treatment or larval age, laminarin-degrading enzymes had the highest overall activity. No differences were found between the production of reducing sugars during the degradation of starch and xylan, nor between that for the degradation of carboxymethylcellulose and trehalose. The production of the latter two was the lowest overall (one-way ANOVA, *F*_4,145_=34.65, *P*<0.0001; Fig. 3).

### pH-dependent dynamics in glycosidase activity of *S. purpuratus*

**Fig. 4.**
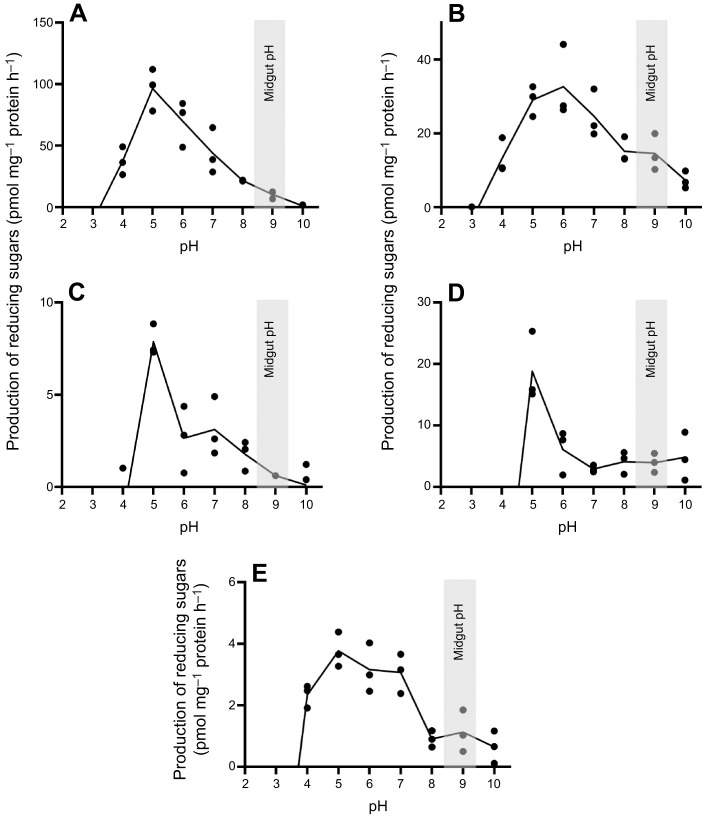
**pH-dependent enzymatic degradation of different carbohydrate substrates in *S. purpuratus.*** Assays were conducted at 30°C; reactions were stopped and the amount of released reducing sugar equivalents detected with DNS. (A) Laminarin, (B) starch, (C) carboxymethylcellulose, (D) xylan and (E) trehalose (*n*=3). Gray bars indicate the pH conditions in the larval midgut (Stumpp et al., 2015).

The pH optimum for the glycolytic degradation of various substrates by crude enzyme extracts was determined by testing the enzymatic activity at different assay pH values spanning a pH range from 3.0 to 10.0. pH optima of the tested enzyme groups were at pH 5.0, with the exception of starch degrading enzymes, which had an optimum at pH 6.0. Values of maximum activity (at pH 5.0) ranged from 96.5±14 pmol mg^−1^ protein h^−1^ for laminarin degrading enzymes up to 3.7±0.5 pmol mg^−1^ protein h^−1^ for trehalose degrading enzymes. Furthermore, enzyme activity displayed a pH optimum for laminarin at pH 5.0 and a nearly linear decrease of production towards pH 10.0, 11% activity at pH 9.0 (Fig. 4A). Starch degradation peaked between pH 5.0 and 7.0, with 45% of the maximal activity at pH 9.0 (Fig. 4B). Cellulose degradation peaked at pH 5.0 with only 8% activity at pH 9.0 (Fig. 4C), and xylan degradation also peaked at pH 5.0, with 21% of the maximal activity at pH 9.0 (Fig. 4D). Trehalose degradation had its optimum between pH 5.0 and 7.0, with only 30% remaining activity at pH values higher than 8.0 (Fig. 4E).

### Thermal profiling of carbohydrate-degrading enzyme activity

**Fig. 5.**
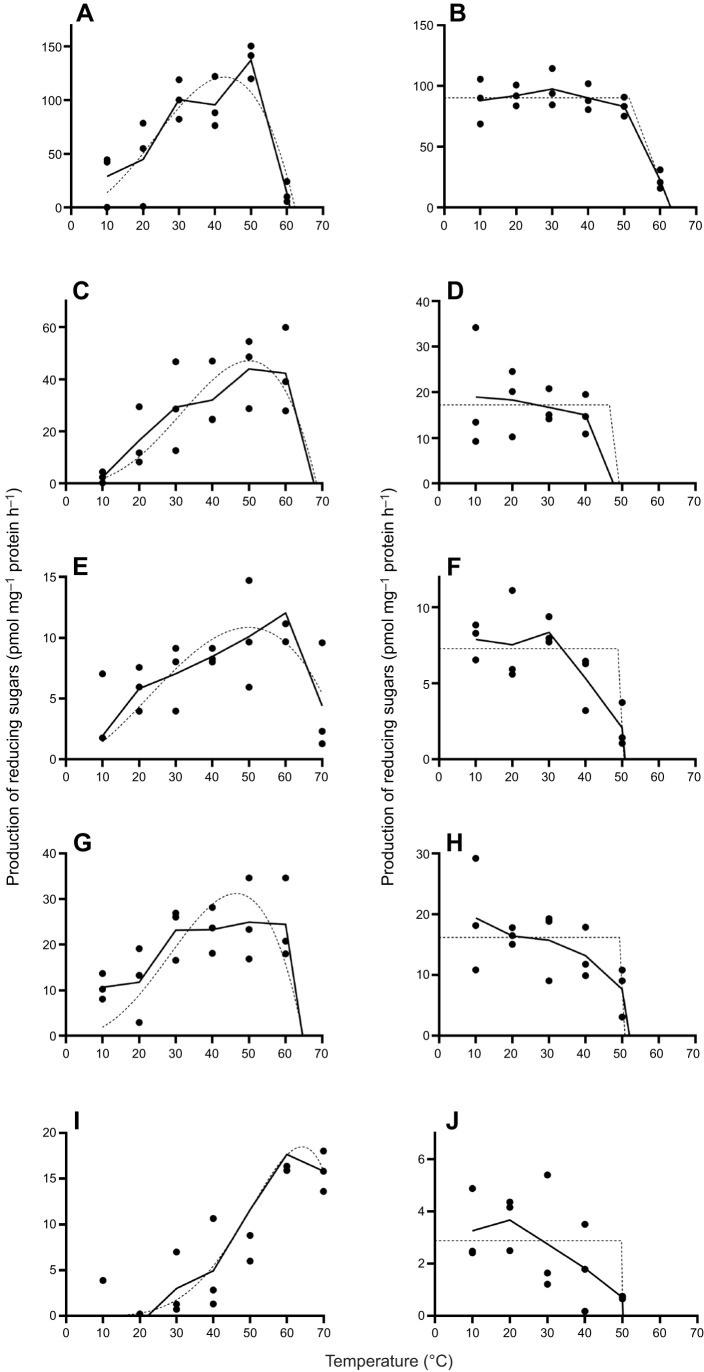
**Thermal profiling of enzymatic carbohydrate degradation in *S. purpuratus.*** For thermal stability analysis, crude enzyme extract was preincubated at the presented temperatures for 1 h before activity was measured at 30°C and pH 5 (or pH 6 for starch); reactions were stopped and the amount of released reducing sugar equivalents detected with DNS. (A) Temperature-dependent laminarin degradation and (B) thermal stability of laminarin degrading enzymes. (C) Temperature-dependent starch degradation and (D) thermal stability of starch degrading enzymes. (E) Temperature-dependent carboxymethylcellulose degradation and (F) thermal stability of carboxymethylcellulose degrading enzymes. (G) Temperature-dependent xylan degradation and (H) thermal stability of xylan degrading enzymes. (I) Temperature-dependent trehalose degradation and (J) thermal stability of trehalose degrading enzymes. *n*=3. Dashed lines are non-linear regression curves.

For all substrates except carboxymethylcellulose and trehalose, there was no enzymatic degradation activity at temperatures above 60°C. With laminarin as a substrate, there was a broad optimum of reducing sugar production spanning from 30 to 50°C. The calculated maximum of 121.5 pmol mg^−1^ protein h^−1^ occurred at 42.6°C (Fig. 5A). Using starch as substrate, there was an increase in the production of reducing sugars with rising temperature between 10 and 50°C. The calculated optimal production with starch degradation reached 47.0 pmol mg^−1^ protein h^−1^ at 50.01°C (Fig. 5C). When employing carboxymethylcellulose as a substrate, there was also an increase in the production of reducing sugars within the temperature range of 10 to 50°C. The computed optimal production with carboxymethylcellulose degradation was 10.9 pmol mg^−1^ protein h^−1^ at 49.88°C. At 70°C, there was still production of approximately 42% with 4.4±3.7 pmol mg^−1^ protein h^−1^ (Fig. 5E). With xylan as a substrate, the production of reducing sugars had a broad optimum between 30 and 60°C. The calculated optimal temperature for xylan degradation was 46.48°C with 31.2 pmol mg^−1^ protein h^−1^ (Fig. 5G). Using trehalose as a substrate, there was a substantial production of 15.8±1.8 pmol mg^−1^ protein h^−1^ of reducing sugars at 70°C. The calculated optimum production with trehalose degradation was 18.5 pmol mg^−1^ protein h^−1^ at 64.13°C, thus notably higher than that for the other substrates (Fig. 5I).

Laminarin-degrading enzymes were stable up to a calculated 51.33°C (Fig. 5B), while starch-degrading enzymes showed stability up to 46.54°C (Fig. 5D). Carboxymethylcellulose-degrading enzymes were stable up to a calculated 48.84°C (Fig. 5F), and xylan-degrading enzymes maintained activity with a calculated production of reducing sugars up to 49.30°C (Fig. 5H). Finally, trehalose-degrading enzymes were calculated to be stable up to 49.82°C (Fig. 5J). Regardless of the temperature applied, pre-incubation of the enzyme extract lowered reducing sugar production when using starch (65% remaining activity) and xylan (84%).

## DISCUSSION

The two most important dietary carbohydrates for the sea urchin larva detected in this study were starch and laminarin. Starch is ubiquitous in phototrophic organisms and it is the most abundant polysaccharide in microalgae, where it is stored intracellularly as a reserve material (Percival and Ross, 1951; Ran et al., 2019). Laminarin constitutes up to 50% of the carbon content in particulate organic matter in plankton samples (Becker et al., 2020).

Based on the enzymatic profiles observed in this study, *S. purpuratus* larvae probably utilize laminarin and starch as primary carbohydrate sources, with laminarinase activity exceeding that of all other glycosidases measured. Expression patterns of *β-1,3-glucosidase* (*laminarinase*) and *α-amylase* candidate genes, along with associated enzyme activity for laminarin and starch breakdown, align with the development of the digestive tract and respond to high food availability through the 4-arm pluteus stage. In *S. purpuratus* larvae, the initial increase in laminarinase activity during gut formation is consistent with that in sand dollar pluteus larvae, where laminarinase activity similarly increases with gut maturation (Vacquier, 1971). The food-dependent increase in amylase candidate gene expression and enzyme activity is comparable to that in the larvae of marine fish (Lu et al., 2018), although the food-induced enzyme activity may be species and substrate specific, as some crustacean larvae exhibited constant enzyme activities, regardless of carbohydrate content in the food (Biesiot and Capuzzo, 1990). However, the enzymatic profiles of this work suggest that laminarin and starch serve as significant dietary polysaccharides for the nutrition and growth of *S. purpuratus*, comparable to *S. intermedius* juveniles directly after metamorphosis (Onitsuka et al., 2015).

Although we found a feed-induced increase in cellulase activity, its peak was 16-fold and 10-fold lower compared to laminarinase and amylase activity, respectively. Furthermore, the food-independent expression of cellulase candidate genes, as well as their low abundance in the single cell transcriptome suggest that cellulases may become more important in adulthood after metamorphosis, when the sea urchin's diet contain cellulose in higher proportions. For example, macroalgae can reach a cellulose content of up to 51% of dry mass in *Gelidium amansii* or 34% of dry mass in seaweeds (Baghel et al., 2021), while its occurrence in *Rhodomonas* sp. is likely to be minimal, because of the lack of a microalgal cell wall. Cellulose is generally considered difficult for most heterotrophic organisms to digest. In many, cellulose is therefore digested with microbial assistance capable of metabolizing carbohydrates, including cellulose – for example, by *Alteromonas* species in shipworms (Goodell et al., 2024) or by *Fusobacteria* (Smith et al., 2017) in the digestive tracts of various adult sea urchin species (Yao et al., 2019). Sea urchin larvae host a specific microbiome whose composition varies with feeding conditions (Carrier et al., 2019). However, a microbial role in the digestive processes of larvae has yet to be verified. Nevertheless, the reorganization of the larval digestive tract during metamorphosis represents an intriguing venture for future research. In addition to a compositional change in the microbiome and digestive machinery in response to a major dietary shift, this transition must also be accompanied by a physiological change in pH regulatory machinery – for example, from an alkaline midgut in larvae (Stumpp et al., 2015) to neutral conditions in adult sea urchins (Jangoux and Lawrence, 1982) – which could also be relevant to digestive processes.

In this work, the patterns of the candidate gene expression and enzyme activities of xylanase and trehalase did not coincide with digestive tract development or feeding treatment. Thus, they cannot be as easily interpreted as being dominantly involved in larval digestion. *Trehalase* transcript levels were 63 times higher under low food conditions, and trehalase activity in larvae under low food conditions was significantly higher. Trehalase influences digestive activity in several species, including insects, mussels and sea cucumbers (Huo et al., 2018; Shang et al., 2022; Teo and Sabapathy, 1990; Terra and Ferreira, 1981). When starvation was used as a stressor in *Caenorhabditis elegans* (Hibshman et al., 2017) and several insects (Tang et al., 2014; Shukla et al., 2016; Shi et al., 2017), trehalase was suggested to be part of a stress-coping mechanism related to nutrient scarcity. In these cases, nutrient scarcity led to increased trehalase activity or *trehalase* expression, similar to what was observed in our study. Studies on the role of trehalose in larval nutrition and energy homeostasis in echinoderms are scarce. However, given our data, we postulate that trehalase may also play a role in dietary stress tolerance in sea urchin larvae. As larval density in our set-up was high, up to 12 larvae ml^−1^, to ensure sufficient material for intensive mRNA and enzyme sampling, it is possible that larvae in both feeding treatments were food limited, albeit to different degrees. This would explain the lack of significant differences in larval size between these two conditions, despite significant effects on digestive enzyme activity and transcript abundance of putative genes between the two treatments.

Slightly acidic pH optima of the investigated glycosidase activities are comparable to those of other invertebrates (Gutierrez et al., 2017 preprint; Li et al., 2020; Rahman et al., 2014; Padilla-Hurtado et al., 2012; Shukla et al., 2016). Optimal pH values for the various glycosidases fall within the range 5.0–6.0 which is also in line with findings for other sea urchins, such as *S. nudus* (Nakatani and Kobayashi, 1996), *Dendraster excentricus* (Vacquier, 1971) and marine crustaceans (Allardyce and Linton, 2008; Crawford et al., 2005). Consequently, the rate and efficiency of digestive processes in sea urchin larvae are probably dependent on gastric pH as has been suggested for total proteinase activity in response to ocean acidification (Stumpp et al., 2013). However, currently, we cannot verify a causal relationship between midgut pH and *in vivo* digestion rates (Hildebrand et al., 2023). Despite the current lack of causal explanation, the glucosidase optima of pH 5.0–6.0 demonstrated here is far from the alkaline conditions in the larval midgut (Stumpp et al., 2015). The considerable loss of activity at a pH of 9.0 (with 11% residual activity for laminarin, 44% for starch, 8% for cellulose, 21% for xylan and 30% for trehalose degradation) still suggests that glycosidases may not operate at maximum capacity under physiological conditions. Again, physiological aspects in the transition from the larval to the juvenile/adult gut is intriguing considering that the carbohydrate content of the adult diet increases, and the midgut pH of adults is reduced to pH ranges that are probably better suited to carbohydrate digestion.

When comparing data from gene expression analysis and enzyme activity, it is important to note that the function of a putative gene may not solely correspond to its actual role within the living species. This discrepancy is a major issue in biochemistry and physiology and poses a significant challenge when attempting to correlate gene expression with enzyme activity, because often enzyme activity does not reflect or correlate with the respective gene expression, potentially as a consequence of post-translational modification (Frye et al., 2018) or compartmentalization in tissues (Pollard et al., 2022). The challenge extends beyond merely inferring function from DNA sequencing to the more complex task of validating these functions through functional genomics. While advances in annotation through deep learning models (Kim et al., 2023) and profiling chromosome structure (Tang, 2023) have provided insights into gene organization and regulatory roles, empirical testing remains essential to confirm these predicted functions. Systems such as the Ensembl gene annotation, which has been applied across over 70 different vertebrate species (Aken et al., 2016), and initiatives such as the FAANG Consortium, which aims to generate comprehensive functional genomic data for farmed animals (Giuffra et al., 2019), highlight the ongoing efforts to improve the accuracy and scope of functional annotation, but still rely on functional genomic approaches to verify the function in living organisms. In the present study, we used qPCR for analysis of putative digestive enzyme genes for elucidating a potential role of these genes in larval digestion and concluded that they may be involved in digestion, when the gene expression was (i) increasing during functional development of the midgut, (ii) detected in midgut cells in the single cell transcriptome, and (iii) responded to high food treatment in the larva, e.g. a higher expression under high food treatment. However, a proof of function using functional genomics of the selected genes in larval digestion is a task for future studies.

### Conclusion

In conclusion, our study provides novel insights into the gene expression and enzymatic activities underlying carbohydrate digestion in sea urchin larvae, and sheds light on their responses to environmental cues. Through examination of the expression of putative glycosidases and corresponding enzymatic activities, we elucidated that larvae of *S. purpuratus* are capable of degrading all polysaccharides investigated in this work, even though some, such as cellulose and xylan, are present only in small amounts in the food source or may have regulatory functions (e.g. trehalase). Interestingly, the comparably high digestive capacity of laminarin in sea urchin larvae suggests that laminarin may be an important molecule as a carbon source not only in benthic but also in planktonic communities (Becker et al., 2020). Furthermore, the digestive tract transition from larval to juvenile/adult system during metamorphosis is an exciting research topic for the future.

## Supplementary Material

10.1242/jexbio.250125_sup1 Supplementary information

Table S1. Statistical analyses. (A) t-test analyses of mRNA expression (manuscript figure 1A, C, E, G, I), (B) t-test analyses of enzymatic characterization (manuscript figure 1B, D, F, H, J), (C) one-way ANOVA followed by Tukey's multiple comparisons test of mRNA expression (manuscript figure 2), (D) one-way ANOVA followed by Tukey's multiple comparisons test of glycosidase activity over larval development (manuscript figure 3), (E) Nonlinear fit of enzymatic temperature profiles (manuscript figure 5A, C, E, G, I), (F) Nonlinear fit of enzymatic temperature stability (manuscript figure 5B, D, F, H, J), (G) Nonlinear fit and t-tests of larval growth (supplementary Fig. S3A), (H) one-way ANOVA followed by Tukey's multiple comparisons test of feeding rates (supplementary Fig. S5).
