# Supplementary material for: Gene expression and enzyme activity analysis of carbohydrate digestion in Strongylocentrotus purpuratus larvae
Source: J Exp Biol. 2025 May 8;228(9):jeb250125. doi: 10.1242/jeb.250125 (PMC12079659; doi:10.1242/jeb.250125)
Supplement: Supplementary information [file jexbio-228-250125-s1.pdf]

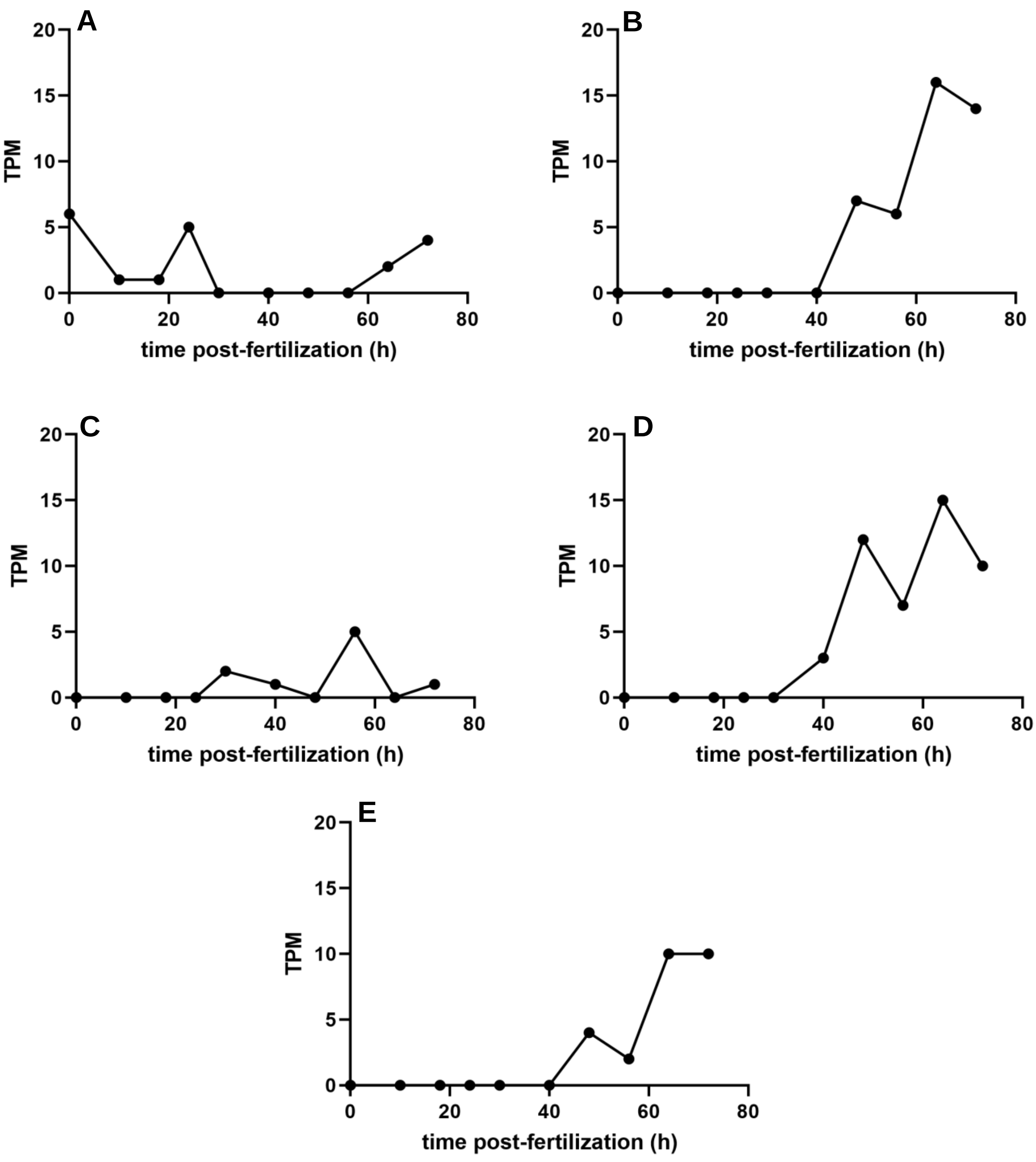

Fig. S1. Expression patterns of candidate genes associated with carbohydrate digestion in *S. purpuratus*. (A) *β-1,3-glucanase (laminarinase) (LOC373274)*, (B) *α-amylase 1 (LOC581100)*, (C) *endo-β-1,4-glucanase D-like (cellulase) (LOC115928076)*, (D) *xylanase/β-glucanase-like (LOC105443845)*, (E) *trehalase (LOC580425)*. Data sourced from (Tu et al., 2014). TPM: transcripts per million.

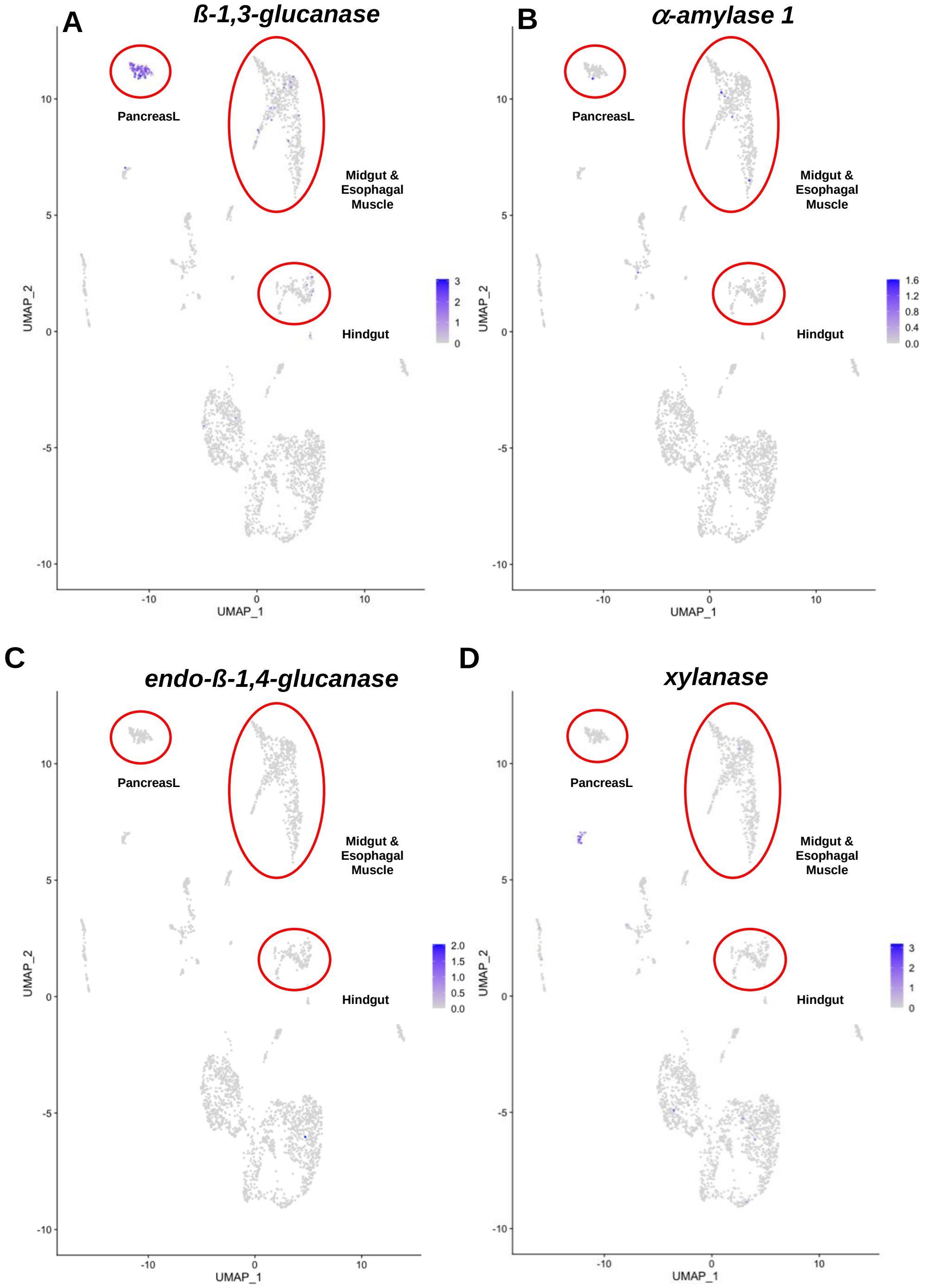
A
ß-1,3-glucanase
PancreasL
Midgut & Esophagal Muscle
Hindgut
UMAP_1
UMAP_2
B
α-amylase 1
PancreasL
Midgut & Esophagal Muscle
Hindgut
UMAP_1
UMAP_2
C
endo-ß-1,4-glucanase
PancreasL
Midgut & Esophagal Muscle
Hindgut
UMAP_1
UMAP_2
D
xylanase
PancreasL
Midgut & Esophagal Muscle
Hindgut
UMAP_1
UMAP_2

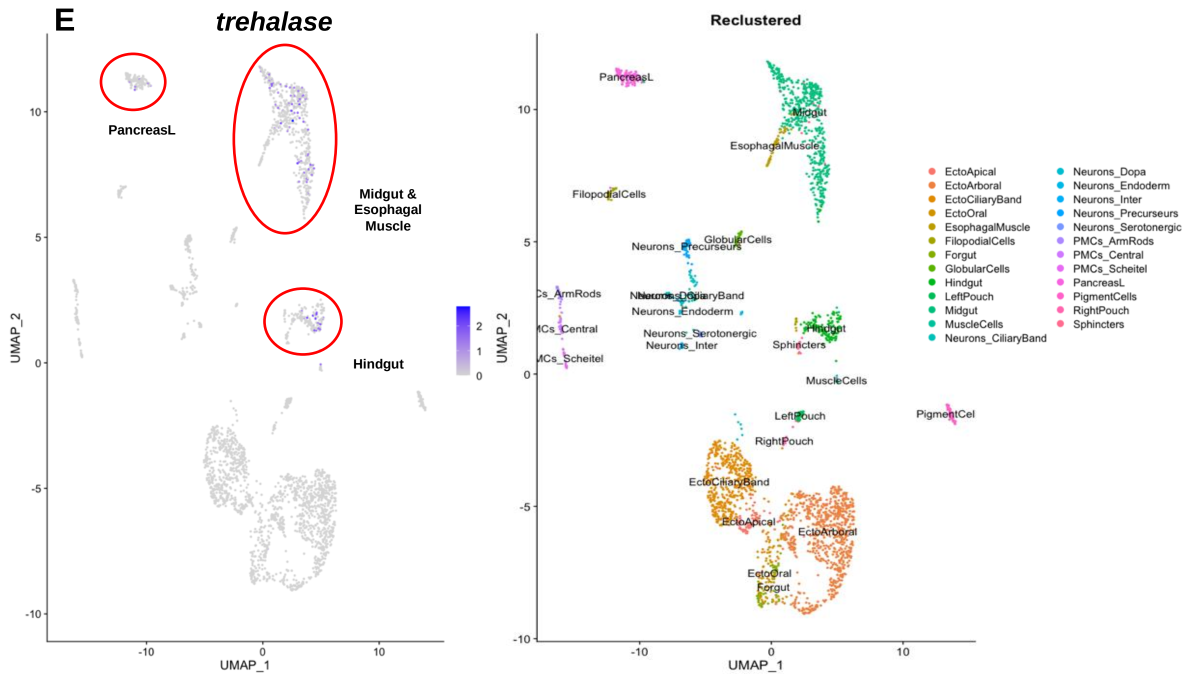

Fig. S2. Single-cell transcriptomic data of candidate genes associated with carbohydrate digestion in *S. purpuratus*. Each point on the 2D graph corresponds to an individual cell. Clusters of points in specific regions exhibit similar gene expression patterns, identifying them as related cell types characterized by known marker genes for those cell types (reclustered graph). Red circles indicate the location of midgut/esophagus, pancreatic like cells and hindgut/intestine on the expression plots (see reclustered plot for all cell types). The depth of the blue color indicates the transcript abundance of the respective gene – darker shades represent higher transcript levels. (A) *β-1,3-glucanase (laminarinase) (LOC373274)*, (B) *α-amylase 1 (LOC581100)*, (C) *endo-β-1,4-glucanase D-like (cellulase) (LOC115928076)*, (D) *xylanase/β-glucanase-like (LOC105443845)*, (E) *trehalase (LOC580425)*. Findings derived from Pluteus larva 72 hours post fertilization.

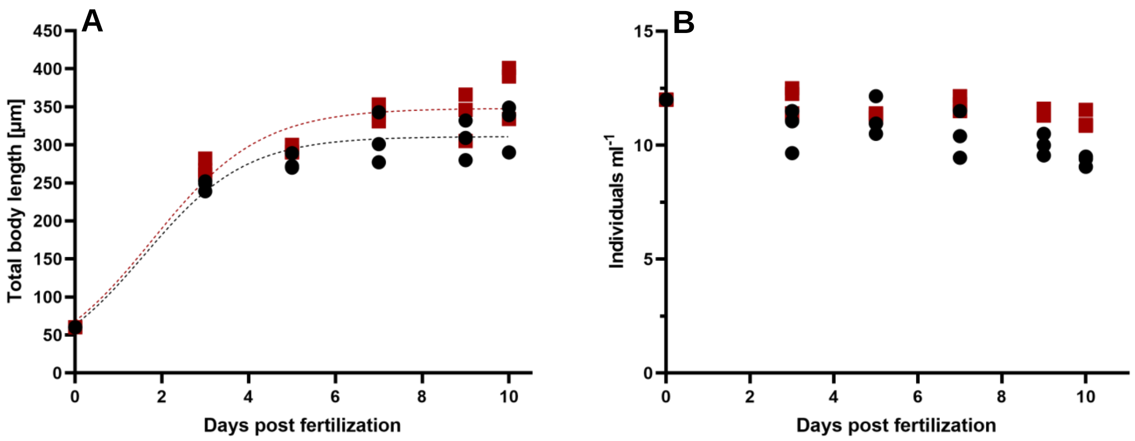

Fig. S3. Characterization of *S. purpuratus* larval cultures. (A) Larval growth and (B) culture mortality under low food (500 cells *Rhodomonas sp.* ml^{-1}, black) and high food (8000 cells *Rhodomonas sp.* ml^{-1}, red), (*n*=3).

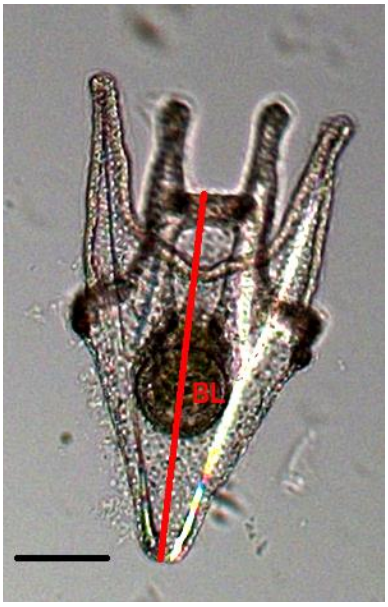

Fig. S4. Morphology of a *Strongylocentrotus purpuratus* 4-arm pluteus larva (10 dpf). BL, body length. Scale bar 75 µm.

Journal of Experimental Biology • Supplementary information

Supplementary Materials and Methods

Larvae were raised according to the procedure outlined in the main manuscript. In brief, spawning was initiated by gently agitating adult sea urchins. The resulting eggs were collected in 0.2 µm filtered seawater (FSW, 31.5 psu) and briefly rinsed with FSW prior to fertilization. Dry sperm was collected and fertilization was achieved by mixing diluted sperm with the eggs. Healthy zygotes with fertilization rates exceeding 99% were transferred to 2 L culture flasks at an initial density of approx. 12 larvae ml^{-1}. Cultures were maintained under low light conditions with a 12-hour light/dark cycle at a temperature of 15°C, and gently mixed using a stream of pressurized air bubbles. Larval health was assessed visually every two days by assessing larval body length (3 larvae per culture, Fig. S3A, Fig. S4) and culture density using two 10 ml samples from each replicate (Fig. S3B). Water was changed every other day, and larvae were fed every other day following the water change, beginning 3 days post-fertilization. Six different concentrations (*n*=3) of *Rhodomonas* sp. were used for characterization of larval growth performance in response to food (0, 500, 2,000, 4,000, 8,000 & 10,000 cells ml^{-1}).

Larval feeding rates were determined by the number of residual cells in larval cultures using a Millipore Guava EasyCyte HT Flow Cytometer and the GuavaSoft 2.7 software (Merck, Darmstadt, Germany). For this purpose, on post-feeding days (6, 8, 10, 12 dpf) larval cultures were thoroughly stirred and 10 µl of culture medium was taken and loaded onto a 96-well plate (three biological replicates). The subsequent workflow was carried out in duplicates for 60 seconds after a 3-second mix. As a reference, 10 µl of Guava easyCheck Kit were combined with 190 µl Guava Check Diluent. The number of cells consumed by the larvae was determined by the difference between the cells added to the cultures (0, 500, 2,000, 4,000, 8,000 and 10,000 *Rhodomonas* sp. ml^{-1}) and the residual cells.

Results

Larvae consumed more algal cells with increasing supply. Larvae reared under conditions of 10,000 cells ml^{-1} consumed 6,658±1,633 cells d^{-1} (equivalent to

Journal of Experimental Biology • Supplementary information

67±16% of the provided food), which was significantly more than larvae reared under conditions of 0, 500, 2,000, and 4,000 cells ml^{-1} ($F_{(5,12)}$=7.993, P<0.0014). Larvae reared under conditions of 8,000 cells ml^{-1} consumed an average of 5,128±1,863 cells d^{-1} (corresponding to 65±23% of the provided food) equivalent to 1.8 times the number of cells d^{-1} compared to larvae reared at 4,000 cells ml^{-1}. Larvae reared under conditions of 500 (78±16% of the provided food), 2,000 (70±21% of the provided food), and 4,000 (71±19% of the provided food) cells ml^{-1} had no significant differences in daily feeding rates (Fig. S2). The average larval density of all cultures did not differ from each other (one-way ANOVA, $F_{(5,\ 138)}$=1.848, P=0.1075).

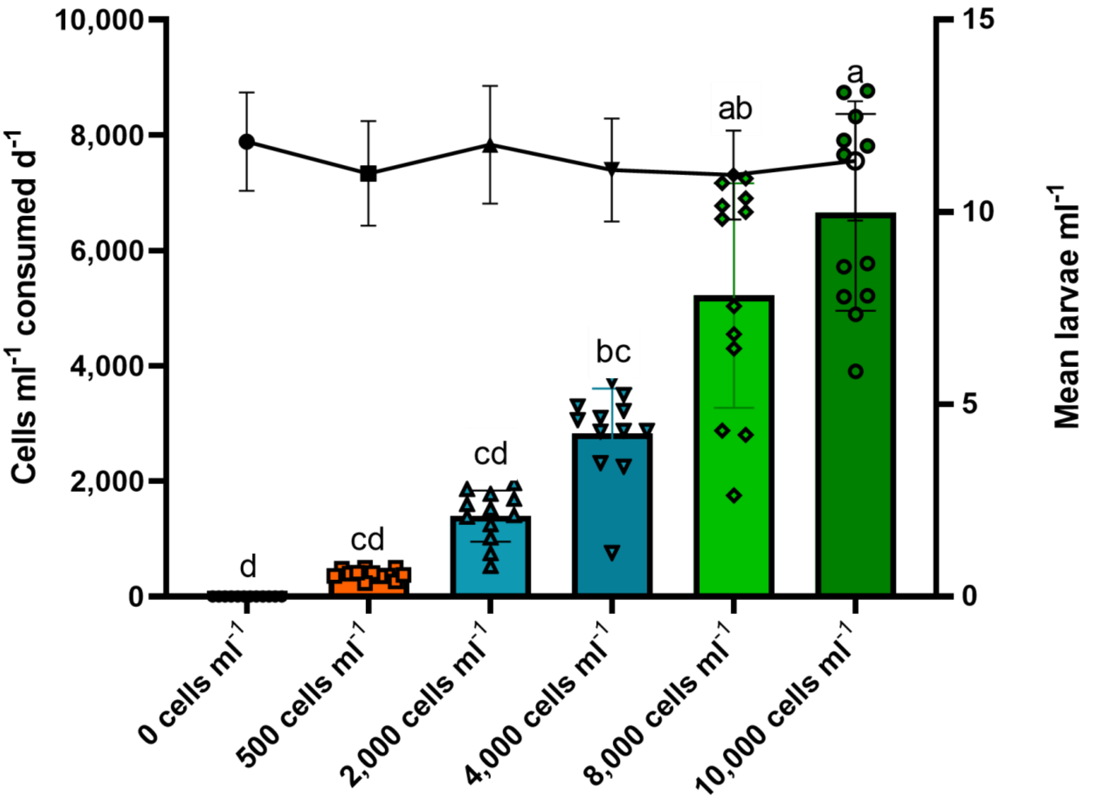

Fig. S5. Feeding rates (bar charts) and total culture density (line) of *S. purpuratus* larvae under different food conditions. Larvae, raised for 12 days under the respective food treatments (approx. 12 larvae ml^{-1}) were administered 0 (light brown), 500 (dark brown), 2,000 (light blue), 4,000 (dark blue), 8,000 (light green) or 10,000 (dark green) cells of *Rhodomonas* sp. ml^{-1} larval culture, (n=3). Different letters indicate statistically significant differences (one-way ANOVA, $F_{(5,\ 66)}$=69.72, P<0.0001).

Journal of Experimental Biology • Supplementary information

Table S1. Statistical analyses. (A) t-test analyses of mRNA expression (manuscript figure 1A, C, E, G, I), (B) t-test analyses of enzymatic characterization (manuscript figure 1B, D, F, H, J), (C) one-way ANOVA followed by Tukey's multiple comparisons test of mRNA expression (manuscript figure 2), (D) one-way ANOVA followed by Tukey's multiple comparisons test of glycosidase activity over larval development (manuscript figure 3), (E) Nonlinear fit of enzymatic temperature profiles (manuscript figure 5A, C, E, G, I), (F) Nonlinear fit of enzymatic temperature stability (manuscript figure 5B, D, F, H, J), (G) Nonlinear fit and t-tests of larval growth (supplementary Fig. S3A), (H) one-way ANOVA followed by Tukey's multiple comparisons test of feeding rates (supplementary Fig. S5).

Available for download at
https://journals.biologists.com/jeb/article-lookup/doi/10.1242/jeb.250125#supplementary-data

References

Tu. Q., Cameron, R. A., & Davidson, E. H. (2014). Quantitative developmental transcriptomes of the sea urchin Strongylocentrotus purpuratus. *Developmental Biology*, *385*(2), 160–167. https://doi.org/https://doi.org/10.1016/j.ydbio.2013.11.019
